# Microchip-based ultrafast serodiagnostic assay for tuberculosis

**DOI:** 10.1038/srep35845

**Published:** 2016-10-24

**Authors:** Vigneshwaran Mani, Bhairav Paleja, Karima Larbi, Pavanish Kumar, Jo Ann Tay, Jie Yee Siew, Fatih Inci, ShuQi Wang, Cynthia Chee, Yee Tang Wang, Utkan Demirci, Gennaro De Libero, Amit Singhal

**Affiliations:** 1 Singapore Immunology Network, Agency for Science, Technology and Research (A*STAR), Singapore; 2 Demirci Bio-Acoustic-MEMS in Medicine (BAMM) Laboratory, Stanford University School of Medicine, Department of Radiology, Canary Center at Stanford for Cancer Early Detection, Palo Alto, California, USA; 3 State Key Laboratory for Diagnosis and Treatment of Infectious Diseases, First Affliliated Hospital, College of Medicine, Zhejiang University, Hangzhou, China; 4 Collaborative Innovation Center for Diagnosis and Treatment of Infectious Diseases, Hangzhou, China; 5 Institute for Translational Medicine, Zhejiang University, China; 6 Tuberculosis Control Unit (TBCU), Tan Tock Seng Hospital, Singapore; 7 Department of Biomedicine, University of Basel, Switzerland

## Abstract

Access to point-of-care (POC), rapid, inexpensive, sensitive, and instrument-free tests for the diagnosis of tuberculosis (TB) remains a major challenge. Here, we report a simple and low-cost microchip-based TB ELISA (MTBE) platform for the detection of anti-mycobacterial IgG in plasma samples in less than 15 minutes. The MTBE employs a flow-less, magnet-actuated, bead-based ELISA for simultaneous detection of IgG responses against multiple mycobacterial antigens. Anti-trehalose 6,6′-dimycolate (TDM) IgG responses were the strongest predictor for differentiating active tuberculosis (ATB) from healthy controls (HC) and latent tuberculosis infections (LTBI). The TDM-based MTBE demonstrated superior sensitivity compared to sputum microscopy (72% *vs.* 56%) with 80% and 63% positivity among smear-positive and smear-negative confirmed ATB samples, respectively. Receiver operating characteristic analysis indicated good accuracy for differentiating ATB from HC (AUC = 0.77). Thus, TDM-based MTBE can be potentially used as a screening device for rapid diagnosis of active TB at the POC.

The management and control of tuberculosis (TB) still remains a significant threat to public health^1^, partly due to the absence of cost-effective, sensitive, and rapid diagnostic tests^2^,^3^. Currently, sputum smear microscopy is the most commonly used point-of-care (POC) method for TB diagnosis in endemic countries, despite its poor sensitivity (30–60%)^4^. Although “gold standard” bacterial culture does provide the required sensitivity, the test takes several weeks and requires well-equipped laboratories and trained staff 5. Such a long turn-around time often results in delayed diagnosis, continued transmission, and the risk of developing drug resistance^6^.

Serological tests based on the detection of antibodies against mycobacterial protein antigens in the form of lateral flow devices or standard ELISAs have been extensively used for the diagnosis of TB^7^. However, these tests have demonstrated poor sensitivity (1–60%) and specificity (53–99%) compared with standard culture methods^8^, performing no better than sputum smear microscopy, and have failed to improve patient outcomes. As such, the World Health Organization (WHO) has recommended against their usage^7^. Endorsement by the WHO of nucleic acid amplification-based TB diagnostic tests, such as the automated GeneXpert MTB/RIF system (Cepheid), INNO-LiPA Rif TB kit (Innogenetics), and Genotype MTBDR*plus* assay (Hain Lifescience) has helped to fill this gap. However, their implementation in POC has been severely restricted by high maintenance costs and the need for sophisticated instrumentation, trained personnel, and uninterrupted electrical supply^9^. Thus, there is an urgent need for the development of a simple, sensitive, and portable assay for the early stage detection of TB at the POC. An ideal test must meet minimum specifications outlined by the WHO, such as short assay time (<3 h), minimal sample preparation steps, maintenance-free instrumentation, low-cost (<$10 per test), and environmentally acceptable waste disposability^10^,^11^.

Advances in microscale and nanoscale technologies offer feasible approaches for the development of miniaturised POC devices^12^,^13^. Microscale technologies allow integration and automation of multistep assays, such as ELISA^14^, thus enabling sample processing, target capture, and detection into a single integrated device, which speeds up the whole assay. In particular, magnetic beads (MB) have been exploited extensively in microfluidic-based ELISAs due to their uniform size, high surface-to-volume ratio, faster reaction kinetics, and ease of manipulation, providing better sensitivity with shorter assay time compared to conventional flat surfaces^15^,^16^. Furthermore, with the use of an external magnet, MBs can be actuated/manipulated^17^,^18^,^19^,^20^ through a series of stationary reagents for bio-detection in automated assays^21^,^22^,^23^,^24^. This provides a simple ‘sample-in and answer-out’ based system, which is highly desirable for diagnosis at the POC.

We present herein the development of a microchip TB ELISA (MTBE), capable of detecting IgG responses against multiple antigens from plasma samples of active TB (ATB) patients in a rapid and miniaturised detection system. The MTBE utilises a *Mycobacterium tuberculosis (Mtb*) surface glycolipid *i.e.* trehalose 6,6′-dimycolate (TDM) and two purified proteins, 38 kDa glycolipoprotein and antigen 85A (Ag85A), as antigens based on their known immunogenicity and their application in TB serodiagnosis^25^,^26^,^27^,^28^,^29^,^30^,^31^,^32^. The MTBE relies on the actuation of antigen-coated MBs through sequentially organised reagents for capturing *Mtb* antigen-specific IgG from the plasma, followed by labelling and colorimetric detection. We showed that MTBEs featuring detection of anti-TDM IgG response could reliably differentiate ATB patients from healthy controls (HC). Furthermore, the test requires less than 15 min from sample addition to detection, which is within the minimum specifications required for POC TB testing^10^.

## Results

### Characterisation of TDM-coated magnetic beads

We first standardized the coating of TDM on magnetic beads. Since high lipid concentrations lead to the formation of large bead aggregates^33^. We assessed different TDM concentrations per surface area on MBs (0.16 μg/cm^2^, 0.41 μg/cm^2^, and 0.65 μg/cm^2^) for optimal TDM coating. The resultant TDM-coated MBs were then tested for size distribution, amount of bound TDM, and the ability to detect anti-TDM IgG using a pooled plasma sample of ATB individuals.

Dynamic light scattering (DLS) showed uniform size distribution of MBs irrespective of TDM surface concentrations (Supplementary Fig. S1). A polydispersity index (PI) of <0.21 was obtained for all the MB preparations, indicating the presence of monodisperse MBs (based on the criteria, PI < 0.3 for monodisperse beads^34^). The mean diameter of the TDM-coated MBs obtained by DLS ranged from 4.2–4.6 μm, which was well within the range of uncoated nascent MBs (dia. 4.5 μm). Furthermore, large multi-bead aggregates were not visible by light microscopy, indicating the monodisperse nature of the TDM-coated MBs (Supplementary Fig. S2).

To assess the amount of TDM adsorbed onto the MB surface, TDM bound to each of the MB preparations was extracted for thin layer chromatography (TLC) analysis. TLC analysis indicated that the recovery of bound TDM (%) from MBs prepared using surface TDM concentrations of 0.16 μg/cm^2^ and 0.41 μg/cm^2^ were similar, but recovery from MBs using concentrations of 0.65 μg/cm^2^ was lower (Supplementary Fig. S3). As higher lipid losses due to nonspecific adsorption to the reaction vials were significant in small batch preparations (0.2 mL), we repeated the TDM recovery process using MBs prepared in a large batch (1.6 mL). In the large batch preparation, MBs with a surface TDM concentration of 0.41 μg/cm^2^ showed 54% recovery of bound TDM. At this concentration and based on the molecular weight of TDM (~2636 g/mol) and the total number of beads used in the preparation, the number of TDM molecules per bead was estimated at ~3.2 × 10^7^. This is in concordance with the theoretical estimate of 3.4 × 10^7^ of TDM molecules per bead based on the surface area of the bead (6.4 × 10^11 ^m^2^, diameter 4.5 μm) and the molecular exclusion area of TDM (~180 °A^2^/molecule), indicating a uniform monolayer of TDM on the bead surface.

Next, we tested the ability of TDM-coated MBs to detect an anti-TDM IgG response in pooled plasma of ATB individuals using a flow cytometry-based MB immunoassay. Bead preparations with a surface TDM concentration of 0.41 μg/cm^2^ displayed the highest levels of anti-TDM IgG antibodies in comparison to MBs prepared using 0.16 and 0.65 μg/cm^2^ of TDM (Supplementary Fig. S4). Since temperature variation and reagent storage may influence the performance of an ELISA, we then tested the stability of TDM-coated MBs (0.41 μg/cm^2^). The beads maintained 95% activity after 5 months of storage at room temperature and 74% activity after 10 months with a relative variation of 65–125% over time (Supplementary Fig. S5). Based on the aforementioned analysis, we selected the MB preparation with a surface TDM concentration of 0.41 μg/cm^2^ for further use.

### Comparison of magnetic bead (MB) ELISA with conventional plate ELISA format for anti-TDM response detection

**Figure 1:**
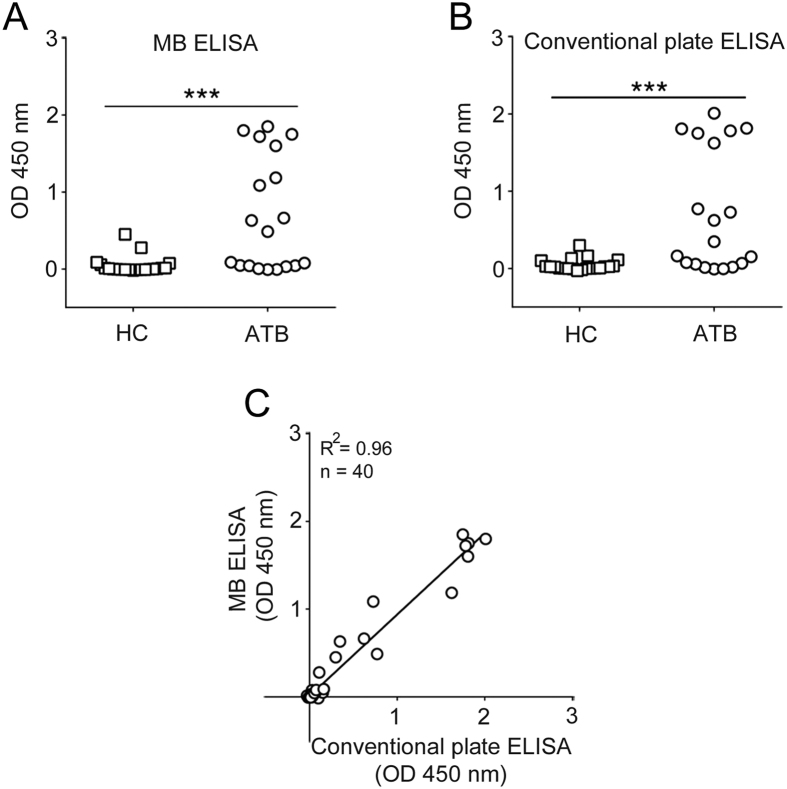
Performance of TDM based magnetic bead (MB) ELISA with conventional plate bound assay. Comparison of the distribution of anti-TDM IgG responses in the plasma of active TB (ATB) and healthy control (HC) individuals determined using (**A**) MB ELISA where plasma was diluted 125-fold in 5% BSA buffer. (**B**) Conventional plate ELISA where plasma was diluted 2500-fold in 5% BSA. N = 40, ATB = 19 and HC = 21 (***P = 0.0003 for MB ELISA; ***P = 0.0007 for plate ELISA). (**C**) Correlation of anti-TDM plasma IgG responses using the MB ELISA and a conventional plate ELISA.

TDM-coated MBs (0.41 μg/cm^2^) were used to perform a MB ELISA for the detection of anti-TDM IgG in the plasma, and the resultant IgG levels were compared with a conventional plate ELISA format (where TDM is coated on the plate surface). Both formats showed significantly elevated levels of anti-TDM IgG in the plasma of ATB patients compared to HC individuals (Fig. 1A,B). We further tested the specificity of the anti-TDM IgG antibodies using a competitive plate assay, where 10% free trehalose was used to block the antigen-antibody reaction. Out of the 22 ATB plasma samples, 12 showed 50% or more inhibition in IgG binding to TDM using free trehalose, suggesting plasma anti-TDM antibody affinity towards the trehalose moiety of TDM (Supplementary Fig. S6). In the remaining 10 ATB samples, no inhibition by free trehalose was observed, indicating binding of anti-TDM antibodies to epitopes nearby the glycosidic bond between trehalose and mycolic acid^35^,^36^. To benchmark the MB ELISA with a standard plate ELISA, we assessed anti-TDM IgG in the plasma of the same individuals using the two methods. Both methods showed sensitivity >68% (Supplementary Table S1) and a good correlation (R^2^ = 0.96, Fig. 1C). The overall assay time for the MB ELISA was ~50 min, substantially faster than the conventional plate ELISA (5 h).

### Design of microchip TB ELISA (MTBE) assay

**Figure 2:**
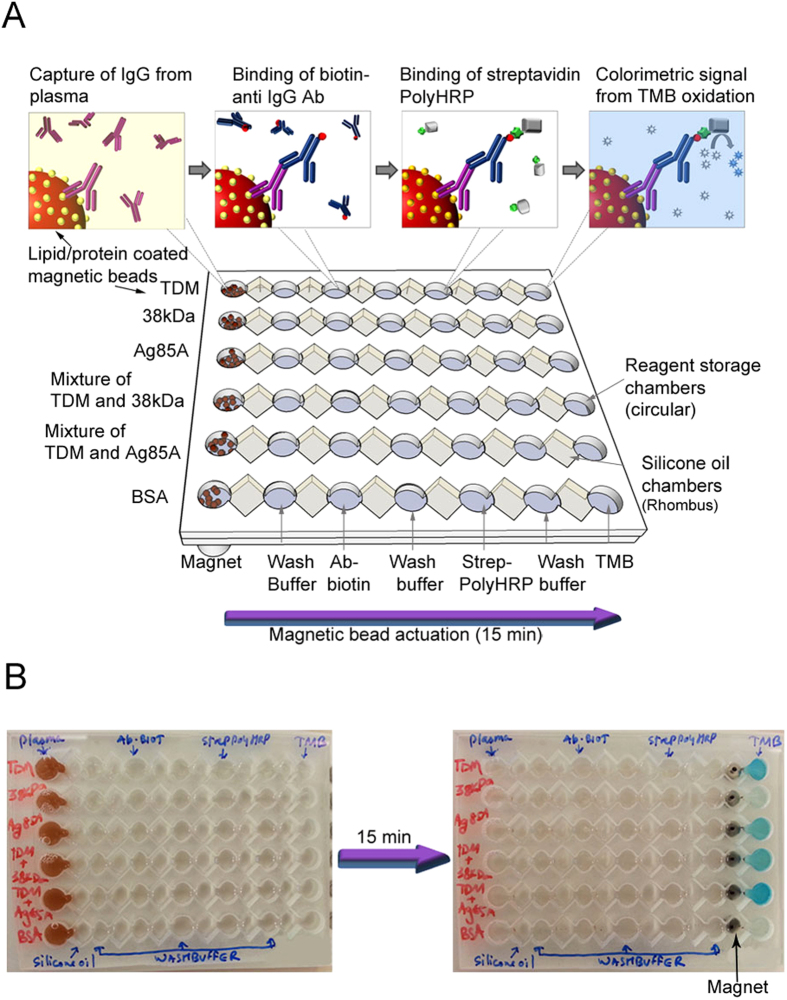
Illustration of MTBE for simultaneous detection of IgG response against multiple *Mtb* antigens for TB diagnosis. (**A**) Schematic representation of a microchip employing magnet-actuated MB ELISA for the simultaneous detection of glycolipid, protein, and a mixture of glycolipid + protein-specific IgG antibodies in the plasma of ATB, latent TB infection (LTBI) and HC individuals. MTBE is performed by simultaneous actuation of antigen-coated MBs in each chamber through sequentially arranged reagents using six magnets below for incubation/washing. The overall time of the test from sample addition to detection is 15 min. (**B**) Photographs of MTBE before (left) and after 15 min (right) from addition of plasma sample.

We then translated the MB ELISA assay onto a microchip device for rapid detection of IgG in plasma samples. Each microchip has six channels in which six different reactions/assays can be performed. Each channel consists of connected chambers, which are filled with spatially arranged, stationary, aqueous reagents separated by immiscible oil. The MTBE is carried out by actuating the antigen-coated MBs in each of these chambers using the magnet underneath. Each chamber performs different functions; the first is used for IgG capture, the second for binding of biotin-labelled secondary anti-IgG antibody, and the third for binding of streptavidin polymeric enzyme, with alternate chambers for washing (Fig. 2A). Finally, the MB-bound polymeric enzyme induces TMB oxidation in the latter chamber, generating a blue-coloured substrate (Fig. 2B). The reaction was stopped and the optical density (OD) was measured at 450 nm. In the first three channels of the MTBE, we detected IgG against TDM, 38 kDa, and Ag85A antigens, respectively. The fourth and fifth channels were used to measure the total IgG response against TDM combined with each protein antigen. The sixth channel was used as negative control with no antigen added. The entire MTBE process requires ~15 min from plasma sample addition to colorimetric detection (Fig. 2B).

### Simultaneous detection of IgG antibodies against multiple *Mtb* antigens using MTBE

**Figure 3:**
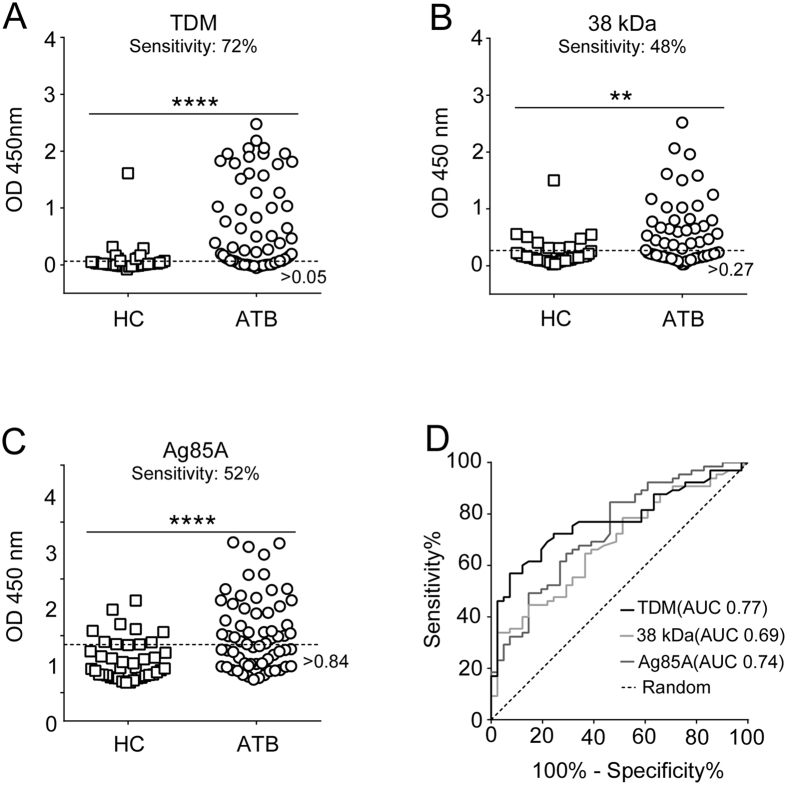
Distribution of IgG responses determined by MTBE against independent antigens in the plasma of ATB and HC individuals. Antigens included (**A**) TDM, (**B**) 38 kDa, (**C**) Ag85A. The plasma in the MTBE was diluted 200-fold in 5% BSA. N = 106, ATB = 65, and HC = 41 (**P = 0.0012, ****P < 0.0001) (**D**) ROC curves for plasma IgG assays for individual antigens for differentiating ATB from HC individuals.

We then used MTBE to detect IgG against three *Mtb* antigens (TDM, 38 kDa, and Ag85A) and their combinations in 146 plasma samples (65 ATB, 40 LTBI, and 41 HC). ATB samples were considered positive if the values were higher than the cut-off values obtained from the HC and LTBI samples^31^. The cut-off value for each antigen was set at 75% specificity in order to compare their relative sensitivity to a set specificity. Using the specificity set criteria, 88% (57/65) of the ATB plasma samples were positive for IgG against at least one of the three antigens tested. Only 40% (26/65) of the ATB plasma samples were positive for IgG against any two of the antigens tested individually, and only 28% (18/65) of the ATB plasma samples were IgG positive for all three antigens. These findings suggest a heterogeneous IgG response against the three antigens. Compared to HC plasma samples, ATB samples had significantly higher levels of IgG against all three antigens (Fig. 3A–C), with the highest sensitivity of 72% for TDM (Table 1), indicating greater reliability of the anti-glycolipid humoral response compared to the anti-protein response in differentiating ATB from HC. In addition, receiver operating characteristic (ROC) curve analysis demonstrated a better performance of TDM-based MTBE compared to 38 kDa and Ag85A in discriminating ATB from HC individuals (area under the curve (AUC) of 0.77 vs. 0.69 and 0.74, respectively) (Fig. 3D, Table 1). These results indicate that the IgG humoral immune response to TDM is a promising immunological marker for ATB detection. Comparison of the TDM MTBE with a classical plate ELISA and MB ELISA showed good correlation (R^2^ = 0.90–0.94; Supplementary Fig. S7A,B), suggesting that the TDM MTBE is as efficient as a benchtop ELISA with the important advantage of being performed in ~15 min without the requirement for sophisticated instrumentation.

A similar trend was observed for differentiating ATB from LTBI individuals. Anti-TDM IgG levels provided the highest discrimination sensitivity (71%) when compared to the responses against the 38 kDa and Ag85A protein antigens (Supplementary Fig. S8A–C and Table S2). ROC curves further confirmed the superior accuracy of the TDM-based MTBE (AUC, 0.75) compared with the protein antigen-based assay in differentiating ATB from LTBI individuals (Supplementary Table S2). Indeed, the IgG response against TDM was the strongest predictor for discriminating ATB from HC and LTBI according to a generalised linear model (using logistic regression analysis^37^).

### MTBE IgG responses against a combination of antigens

Next, we investigated whether the collective measurement of the IgG response against two antigen types might further increase MTBE sensitivity. Therefore, we mixed TDM-coated MBs with either one of the protein antigen-coated MBs and performed the MTBE assay as before. The IgG response against the combination of MBs was able to differentiate ATB from HC individuals (Supplementary Fig. S9). However, the sensitivity obtained with combined MBs (65–71%) was no higher than that obtained by measuring response to TDM-coated MBs alone. This finding was confirmed by ROC curve analysis (Table 1). Similarly, the MTBE based on the combined MBs did not show any improvement over TDM MBs alone in discriminating ATB from LTBI individuals (Supplementary Fig. S10 and Table S2).

**Figure 4:**
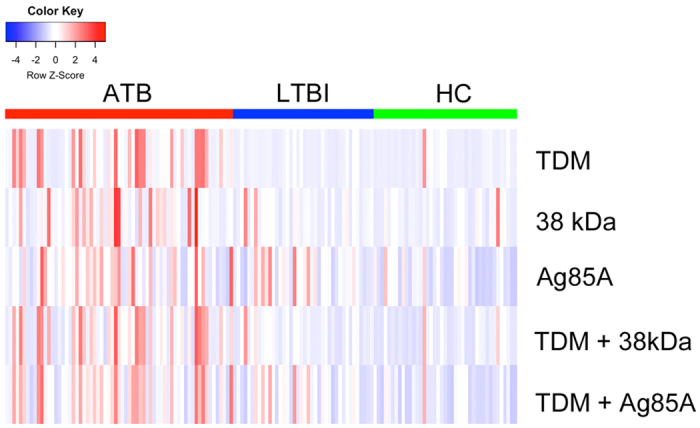
Patterns of reactivity of plasma to antigens in ATB, HC, and LTBI individuals. The heat map shows the reactivity of plasma to individual antigens, and their combinations. Each column represents the response observed in one plasma sample and each row depicts the response to different antigens or their combinations. Normalised OD values (row z-scores) are visualised as a colour spectrum as shown. The heat map was generated using R statistical computing software, using z-score = (x−μ)/σ, where x is an individual’s OD response, μ is mean of OD response from all individuals (N = 146) for each antigen and σ is the standard deviation. N = 146; ATB = 65; LTBI = 40; HC = 41.

We next tested ATB, LTB, and HC plasma samples for the preference of relative IgG responses against each of the three antigens and their combinations. We observed diverse and highly variable antibody patterns, with anti-TDM IgG responses (Normalized OD intensities, z-scores) more clearly differentiating ATB patients from LTBI and HC individuals (Fig. 4).

### Effect of the sputum bacillary load on the IgG antibody response

**Figure 5:**
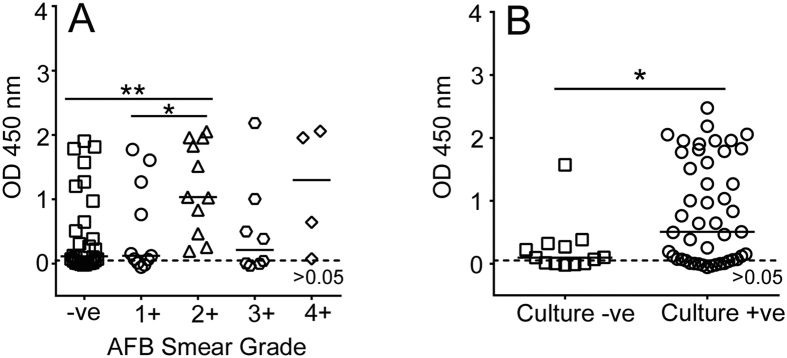
Distribution of anti-TDM IgG response among classified ATB samples. (**A**) Samples were classified based on AFB sputum smear grade. N = 62; -ve = 28; 1+ = 11; 2+ = 11; 3+ = 8 and 4+ = 4 (**P = 0.002 *P = 0.013). (**B**) Samples were classified according to culture test results. Culture positive, N = 47; culture negative N = 13 (*P = 0.0312).

We next compared the anti-TDM IgG levels according to the sputum bacterial burden. The TDM-based MTBE was positive in 80% of sputum smear-positive ATB samples and in 63% of smear-negative ATB samples. As before, the sensitivity of the TDM-based assay was higher than that of the protein-specific assays (Table 2). When ATB samples were stratified according to their smear grade, all smear grade 2+ and 4+ samples were positive for TDM-based IgG responses, with smear grade 2+ samples showing significantly higher anti-TDM IgG responses compared with smear-negative samples (Fig. 5A). Only a few samples from patients with smear grade 3+ were positive for TDM-based IgG responses. In addition, culture-positive ATB samples showed higher TDM-specific IgG responses than culture-negative samples (Fig. 5B). No association of bacillary load with an IgG response to the protein antigens was detected (Supplementary Fig. S11). TDM-specific responses were detected in 77% of culture-positive ATB samples and in 61% of culture-negative ATB samples, and provided a superior correlation with bacterial load compared with antibody responses against the other two tested protein antigens (Table 2). The parameters of the MTBE described here comply with the minimum specifications required for TB POC testing as defined by the WHO, indicating a possible application of the TDM-based MTBE for the early detection of ATB patients (Table 3)^10^,^38^.

We next evaluated the possibility of combining MTBE and sputum microscopy using a two-stage screening process *i.e.* sputum microscopy samples with negative results followed up using the TDM-based MTBE. This combination enhanced the sensitivity of ATB detection from 56% (sputum microscopy alone) to 85% (sputum microscopy in combination with the MTBE), thus demonstrating that the MTBE could be used alongside smear microscopy to improve the accuracy of TB screening.

## Discussion

Despite the identification of *Mycobacterium tuberculosis* as the cause of TB more than a century ago, the diagnosis of TB in resource-limited settings continues to be a major challenge. TB diagnosis in endemic countries still depends primarily on sputum microscopy and culture, which have a number of limitations in terms of sensitivity, specificity, and turn-around time. Here, we present a simple and ultrafast miniaturised ELISA capable of detecting IgG responses against multiple antigens that can be used for the diagnosis of TB. The MTBE provides a simple sample addition (sample-in) to colorimetric detection (answer-out)-based system to differentiate active TB from healthy controls/latent TB infections with results available in less than fifteen minutes.

The sensitivity of TDM-based MTBE (72%) or 38 kDa-based MTBE (46%) is in close agreement to classical plate bound TDM or 38 kDa ELISAs (69% and 47%, respectively)^7^. Moreover, the MTBE test performs well in comparison to other commercial tests that use 38 kDa and TDM as antigens, such as Pathozyme Myco-G (Omega diagnostics, UK) (10–85% sensitivity) and the TB glycolipid assay (Kyowa Medex, Japan) (59–90% sensitivity), respectively^39^. The higher sensitivity of TDM-based MTBE compared to sputum microscopy (72% *vs.* 56%) will allow the rapid screening and diagnosis of individuals with clinically active TB. This enhanced TDM response among active TB individuals is unlikely due to the prior BCG vaccination, since BCG vaccination is mandatory in Singapore and so most likely all subjects enrolled in our study had received it. Noteworthy, it has been suggested that the serological diagnosis using anti-TBGL (Tuberculosis glycolipid antigens) antibody is not influenced by prior BCG vaccination^40^. In addition, the MTBE offers the advantages of being faster, simpler, and cheaper, thus suitable for initial screening of potential TB patients who can later be verified with PCR-based assays.

The MTBE platform has three important technological implementations compared with a conventional ELISA. First, the working principle of MTBE *i.e.* MB actuation through chambers prefilled with stationary reagents circumvents the need for additional equipment, such as expensive pumps, valves, and sample metering that usually require challenging microscale fabrication techniques. MTBE requires simple non-lithographic fabrication methods, and no sophisticated tools are needed to perform the test. Thus, the cost of the test, including reagents and device fabrication, remains extremely low (<U.S. $10). Second, contrary to the conventional ELISA, the MTBE approach utilises polymeric horseradish peroxidase (HRP) labels as opposed to single HRP-labelled secondary antibodies. The higher ratio of HRP to polymer in the labelling step (400 HRP/polymer) amplifies the colorimetric signal and enables detection of low IgG titre samples. Third, the bead-based ELISA assay employed in the microchip has specific advantages over conventional flat surfaces. The high local concentration of antigens bound on the bead surface promotes efficient antigen-antibody binding compared to flat plate surfaces^41^, where binding is facilitated by slow diffusion, and requires several hours to reach saturation. This translates to faster reaction times for each step in the MTBE, significantly reducing the overall time of the assay to ~15 minutes using plasma sample matrices, but still performing as efficiently as the standard classical ELISA. Moreover, the plasma-based testing presented herein is easy to perform because of its simple sample collection (whole blood, venous/finger prick) compared to sputum sample (deep cough, several attempts required). This is particularly relevant for sample collection from children for whom sputum collection is challenging and invasive. Furthermore, the current MTBE assay has many advantages over commercial immunochromatographic (IC) tests, which are widely used for TB diagnosis in low-income high burden countries. Although IC tests are user-friendly, rapid, and affordable, they lack the sensitivity of a classical ELISA (53%, 95% CI 42–64%^39^) and the results are qualitative, relying on a subjective interpretation of aggregated gold nanoparticle band intensity. Furthermore, IC tests are only able to provide binary reports (yes/no) for a single antigen, and analysing responses against multiple antigens is complex. In contrast, the MTBE device represents a simple and robust platform, which is as sensitive as a bench-top ELISA and provides an accurate numerical interpretation of responses against multiple biomarkers.

The MTBE platform is a flexible technology that can be adapted to diagnosis of other diseases. Simultaneous use of multiple rows could even be used to detect multiple biomarkers for several diseases at the same time. The detection mode is not restricted to a colorimetric readout, and can be translated to more sensitive electrochemical^42^, electrochemiluminescent^43^, and plasmonic readouts^44^. The current prototype has the applicability to be further developed into a fully automated device for operation in resource-limited settings. We envisage that the automation of magnet actuation and the integration of a portable colorimetric sensor into a single device presented here will provide a single-step miniaturised assay for TB detection. Such a device will be truly useful in POC TB diagnosis for screening patients and TB contacts, where immediate treatment decisions are of high clinical significance^45^,^46^.

In conclusion, the MTBE platform is the first step towards the development of a POC test for TB and can be easily implemented in combination with sputum microscopy to speed up TB diagnosis in triage and community settings.

## Methods

### Chemicals and Materials

Tosyl-activated superparamagnetic microbeads M-450 (MBs, Dynabeads, Cat. no. 14013, 4.5 μm diameter, 4 × 10^8^ beads/mL, 30 mg/mL) were from Invitrogen. TDM from *Mycobacterium bovis* (Cat. no. T3034), and fatty acid-free bovine serum albumin (BSA, Cat. no. A7030) were from Sigma. Goat F(ab’)_2_ anti-human IgG (H + L) labelled with biotin was from Southern Biotech. Nunc PolySorp 96-well plates, 1-step ultra TMB ELISA, and Pierce streptavidin poly-HRP were obtained from Thermo Scientific. Mycobacterial recombinant 38 kDa and Ag85A proteins were obtained from Mybiosource (CA, USA). Neodymium disc magnets (diameter 5 mm, thickness 2 mm) were from AliExpress Global Retail. Unless otherwise specified, all experiments were performed using PBS buffer without Ca^+2^ and Mg^+2^ ions.

### Human subjects and sample collection

Blood samples of ATB, HC, and LTBI individuals were collected at the Tuberculosis Control Unit, Tan Tock Seng Hospital, Singapore after taking informed consent. Blood plasma was separated in a BSL3 facility, and immediately stored at −80 °C. Of the samples collected, plasma from 65 ATB, 41 HC, and 40 LTBI individuals were randomly selected and stratified based on clinical data, such as the interferon-gamma release assay, sputum smear microscopy, and culture tests. The study was performed in accordance with relevant regulations and was approved by National Health Group domain specific review board (NHG DSRB No. 2010/00566). Sputum samples were stratified based on the AFB smear grade, which was performed according to the American Thoracic Society (ATS) with negative (-ve) representing 0 AFB/100 fields; 1+ representing 1–9 AFB/100 fields; 2+ representing 1–9 AFB/10 fields; 3+ representing 1–10 AFB/field and 4+ representing >10 AFB/field.

### Preparation of lipid and protein-coated MBs

For TDM coating, 0.8 mL of MB stock (4 × 10^8 ^beads/mL) were taken in a glass tube with a screw cap, and magnetically washed sequentially with 70% and 100% ethanol. The MBs were then air dried, and 84 μg TDM in 2.4 mL of solvent (9:1 hexane: ethanol) was added to the dried MBs. The MB dispersion was then sonicated for approximately 1 h in a water bath until the solvent evaporated to dryness. Control beads (beads without TDM) were similarly sonicated with TDM-free solvent, until the solvent evaporated. This step was followed by chemical and physical blocking, whereby dried TDM-coated MBs were sonicated for 2 min with 1.6 mL of 0.1% BSA/PBS buffer, and subsequently sonicated for another 2 min after addition of 6.4 mL of 0.2 M Tris buffer, pH 8.0. The bead dispersion was then mixed in a slow tilt rotor for 24 h at room temperature and washed with 10 mL of 0.1% BSA four times before reconstituting in 1.6 mL of 0.1% BSA/PBS buffer, and stored at 4 °C until further use.

For protein coating, 38 kDa and Ag85A proteins were covalently linked to MBs according to the manufacturer’s protocol (Invitrogen). Briefly, 0.2 mL of stock MBs were washed with 1 mL of 0.1 M borate buffer, pH 9.5. Then, 40 μg of protein per mg MBs were added and allowed to mix in a slow tilt rotor for 30 min. In the next step, BSA was added to final concentration of 0.1% and allowed to mix for additional 24 h. The MBs were washed twice with 0.1% BSA/ PBS, chemically and physically blocked with 0.2 M Tris and 0.1% BSA buffer overnight. MBs were washed twice with 0.1% BSA/ PBS, reconstituted in 0.4 mL of 0.1% BSA, and stored at 4 °C until further use.

### Microchip device fabrication

The microchip was fabricated using a non-lithographic technique as previously reported^47^,^48^. The device consisted of two clear poly(methyl methacrylate) (PMMA) laser cut sheets assembled and bonded. The templates were designed using AutoCAD software. The designs were then supplied to vendor (Ying Kwang Acrylic Trading, Singapore) for laser cutting services. The top template was laser cut on 3-mm thick PMMA, whereas the bottom template was cut on 1.5-mm thick PMMA. The two templates were bonded using spray adhesive (3M, Super 75). The adhesive was allowed to bond and dry for 20 min before the microchip was ready to use. The approximate dimensions of the six-channel microchip device were 95 mm × 70 mm × 5 mm (length × breadth × height).

### MTBE principle

The microchip device consists of six channels with each channel featuring seven circular chambers interconnected alternately with six rhombus chambers. The circular chambers contain 70 μL of aqueous reagents, e.g. lipid/protein-coated MBs, wash buffer, detection tracer antibody, polymeric enzyme labels and colorimetric substrate, whereas the rhombus chambers contain 70 μL of immiscible silicone oil. The oil provides a barrier between the aqueous reagents, and permits manually assisted magnetic actuation of beads between the reagents. Plasma and reagents were diluted in 5% fatty acid free BSA in PBS buffer. In channels 1, 2, and 3, an equal mixture of antigen-coated beads (5 μL, 10^6^ beads) and BSA-coated beads (5 μL, 10^6^ beads) were used, whereas channel 4 and 5 contained equal mixtures of TDM-coated MBs (5 μL) and 38 kDa- or Ag85A-coated MBs (5 μL); channel 6 contained BSA-coated MBs (10 μL, 2 × 10^6^ beads) as a control. In the first step, beads were reacted with 60 μL of plasma (1:200) for 3 min. After the capture of specific IgGs, beads were magnetically actuated to the wash chamber (30 sec) to remove nonspecific plasma proteins. Beads were then actuated to the biotin anti-hIgG (500 ng/mL) chamber for biotin-antibody labelling for 2 min. After brief washing (30 sec), the beads were then actuated to the streptavidin poly-HRP (500 ng/mL) chamber for an additional 2 min for enzyme labelling. After another wash (30 sec), beads were actuated to the one-step ultra TMB substrate solution and incubated for 5 min. The colorimetric reaction was stopped using an equal volume of 2M H_2_SO_4_ and the resultant solution was immediately transferred to a 96-well plate for absorbance measurement at 450 nm using a microplate reader (PerkinElmer Envision 2104 multilabel reader). Absorbance values for the test antigen beads (Channel 1–5) were subtracted from the BSA beads (Channel 6), and the difference correlated to the amount of lipid/protein-specific antibodies present in the plasma. The total time of the microchip ELISA assay is ~15 min.

### Data analysis

The absorbance values for the microchip and conventional plate or MB-bound assay was measured at 450 nm, by subtracting the values of the control BSA-coated MBs from the test antigen-coated MBs. The cut-off point (horizontal dotted line on the figures) and the sensitivity of the assay were determined by maintaining a constant specificity of 75%. This limit was chosen to compare the sensitivity to multiple antigens at a constant specificity. The ROC curves were generated in GraphPad Prism 5 software, by plotting the true positive rate and the false positive rate. Positive predicting values were obtained from the ratio of true positive samples and the sum of true and false positive samples, whereas negative predicting values were obtained from the ratio of true negative samples and the sum of true and false negative samples. Significant differences in IgG responses between different populations were determined using the Mann-Whitney unpaired t-test (GraphPad Prism 5).

## Additional Information

**How to cite this article**: Mani, V. *et al*. Microchip-based ultrafast serodiagnostic assay for tuberculosis. *Sci. Rep.* **6**, 35845; doi: 10.1038/srep35845 (2016).

## Supplementary Material

Supplementary Information

## Figures and Tables

**Table 1:** Evaluation of serodiagnostic potential of each antigen and their combinations.

Active TB vs. Healthy controls					
Antigen	Sensitivity (%)	Specificity (%)	Positive predictive value (PPV) %	Negative predictive value (NPV) %	ROC, AUC
TDM	72	76	82	63	0.77
38 kDa	48	76	76	48	0.69
Ag85A	52	76	77	50	0.74
TDM + 38 kDa	65	76	81	57	0.77
TDM + Ag85A	71	76	82	62	0.80

**Table 2:** Percentage of IgG-positive samples classified according to sputum smear test and culture test.

Antigen	Smear-positive culture confirmed cases (%)	Smear-negative culture confirmed cases (%)	Culture-positive confirmed cases (%)	Culture-negative confirmed cases (%)
TDM	80	63	77	61
38 kDa	46	52	45	69
Ag85A	49	59	51	69
TDM + 38 kDa	66	67	68	61
TDM + Ag85A	77	67	72	77

**Table 3:** Comparison of minimum specifications recommended for POC TB testing^10^.

Features	WHO minimum specifications	Microchip TB ELISA (Current assay)	Sputum microscopy (Conventional technique)	Cepheid’s GeneXpert
Type of assay	NA	Microchip-based *Mtb*-specific glycolipid immune response detection	Direct acid-fast bacilli (mycobacteria) detection	Polymerase chain reaction based detection
Biomarkers	NA	Antibody response to trehalose dimycolate (*Mtb* lipid), to 38 kDa, and to antigen 85A (two *Mtb* proteins)	Whole *Mtb*	Automated system that performs detection of Mtb *rpoB* gene and associated mutations
Sample matrix	NA	Plasma/Serum/Blood	Sputum	Sputum
Time to results	3 h max; desirable 15 min	15 min	2 h	2 hr
Sample preparation	3 steps maximum. -Can be performed in BSL-1 Ability to use small volumes	2-step assay, collect the sample & add to the chip followed by detection -Can be performed in BSL-1	1-step assay, sputum samples are stained and examined under a microscope -Can be performed in BSL-1	-2 step assay -Decontamination of sputum, followed by addition of sample to cartridge
Laboratory infrastructure	NA	No	No	Yes
Throughput	20 tests per day by a single operator	24–32 tests/day by a single operator	>20 tests per day by a single operator	Up to 16 tests per day by a single operator using GeneXpert IV
Waste disposable	Environmentally acceptable disposable as simple burying or burning	Chip burying may be possible	Slides can be buried	NR
Instrumentation	-Maintenance free -Robust in tropical conditions -Acceptable replacement cost -Must fit in backpack, be shock-resistant and work from battery	-Single disposable chip per test - potential device does not require complex instrumentation, thus low replacement cost -potential prototype device can be easily fitted inside a backpack	-Single disposable slides -Yes, requires only a microscope -Not possible to be carried on a backpack	-Maintenance required -Associated replacement cost -Bulky instrumentation
Possibility of POC application at resource limited settings		Yes	Yes	No (GeneXpert can be adopted only at community level as it needs basic laboratory infrastructure)
Cost	Below $10 per test	<U.S. $10 -Low cost of chip -Use of low volume of reagents -Easy to perform	$1 per test -low cost of slides -Microscopes are widely available	$17620 instrument $10–15 per cartridge Needs skilled personnel
Sensitivity (Adult)	Smear positive 95% Smear negative 60–80%	Smear positive 80% Smear negative 63% (ATB vs. HC)	34–60%	Smear positive 98% Smear negative 68% (ATB vs H)
Sensitivity (children)	80%	NP	10–20%	67%
Specificity	95%	75% (ATB vs. HC)	Needs to be confirmed with other methods, cannot differentiate *Mtb* from other mycobacterial strains.	98%

NA: not applicable, NP: not performed, NR: not reported.
