# Supplementary material for: Microchip-based ultrafast serodiagnostic assay for tuberculosis
Source: Sci Rep. 2016 Oct 24;6:35845. doi: 10.1038/srep35845 (PMC5075771; doi:10.1038/srep35845)
Supplement: Supplementary Information [file srep35845-s1.doc]

**Supplementary data for**

**Microchip-based ultrafast serodiagnostic test for tuberculosis**

Vigneshwaran Mani^1^, Bhairav Paleja^1^, Karima Larbi^1^, Pavanish Kumar^1^, Tay Jo Ann^1^, Siew Jie Yee^1^, Fatih Inci^2^, ShuQi Wang^2^, Cynthia Chee^3^, Yee Tang Wang^3^, Utkan Demirci^2^, Gennaro De Libero^1,4*^ and Amit Singhal^1*^

**Supplementary Figures**


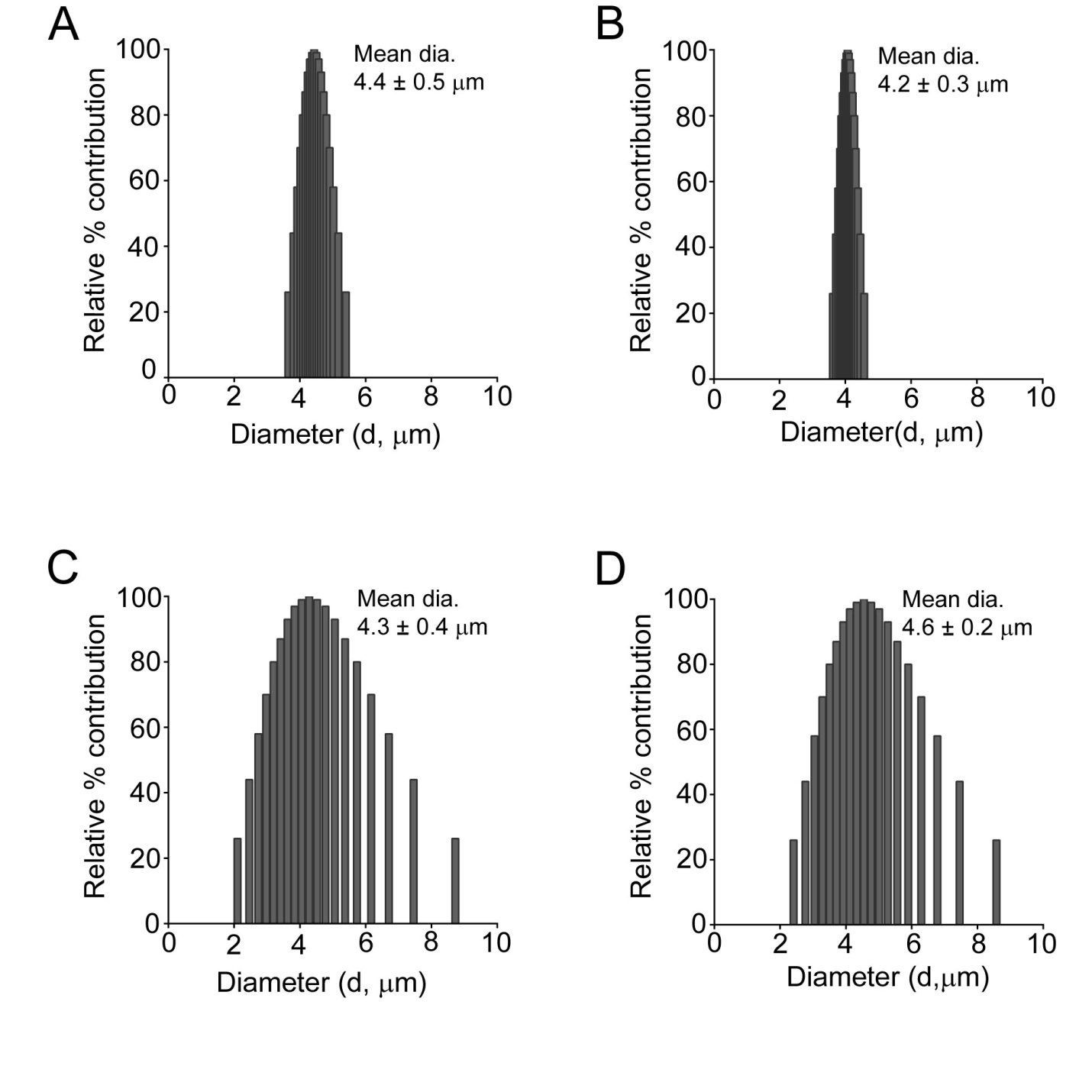


Figure S1. DLS of TDM-coated MB preparations with varying nominal TDM surface concentrations. A) 0 µg/cm^2^, B) 0.16 µg/cm^2^, C) 0.41 µg/cm^2^, and D) 0.65 µg/cm^2^. The mean diameter of each bead preparation was estimated using the Brookhaven particle size analyzer software.


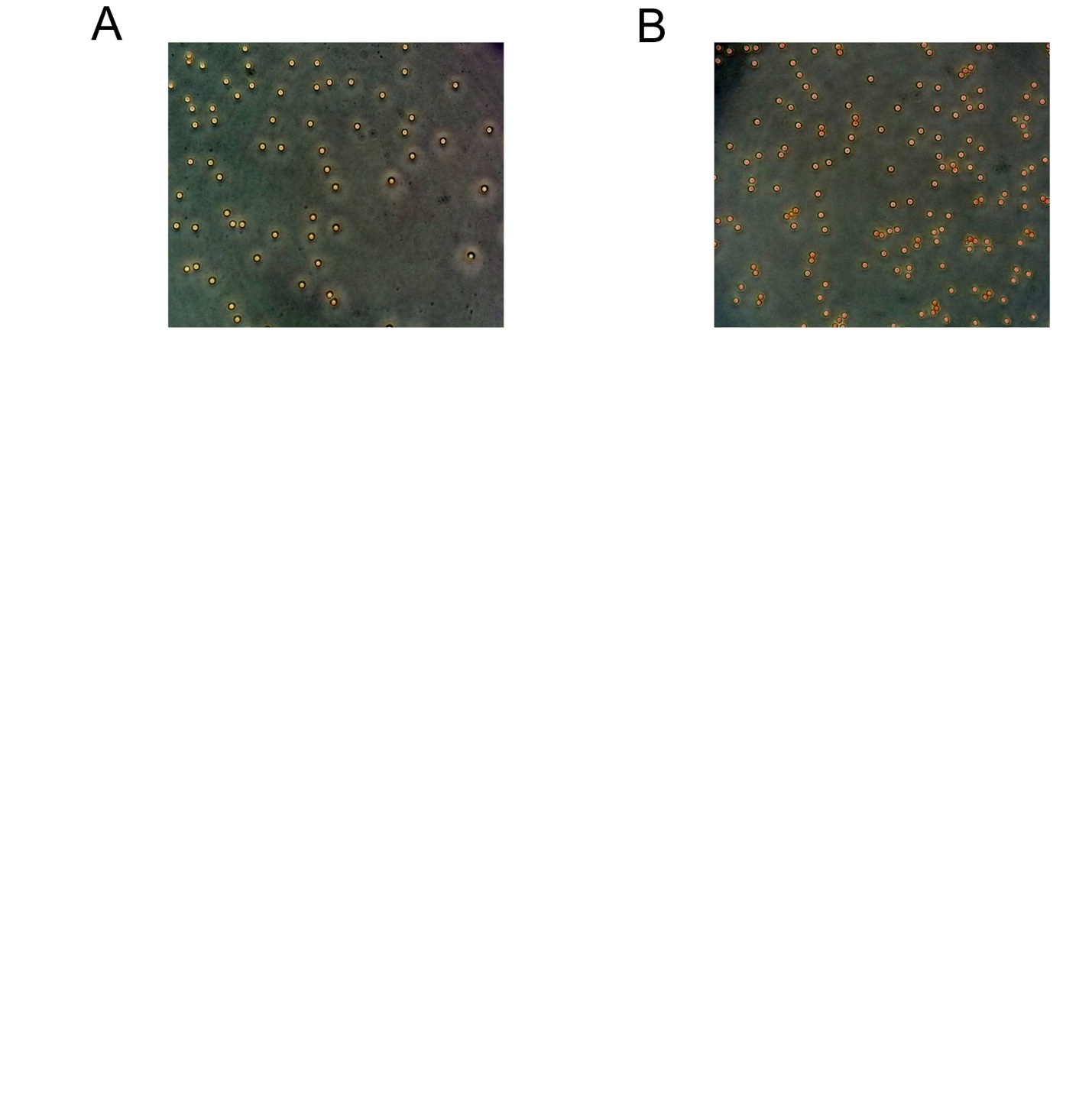


**Figure S2.** Light microscope images of uniform monodisperse A) BSA-coated MBs and B) TDM-coated MBs (0.41 µg/cm^2^), 400× magnification.


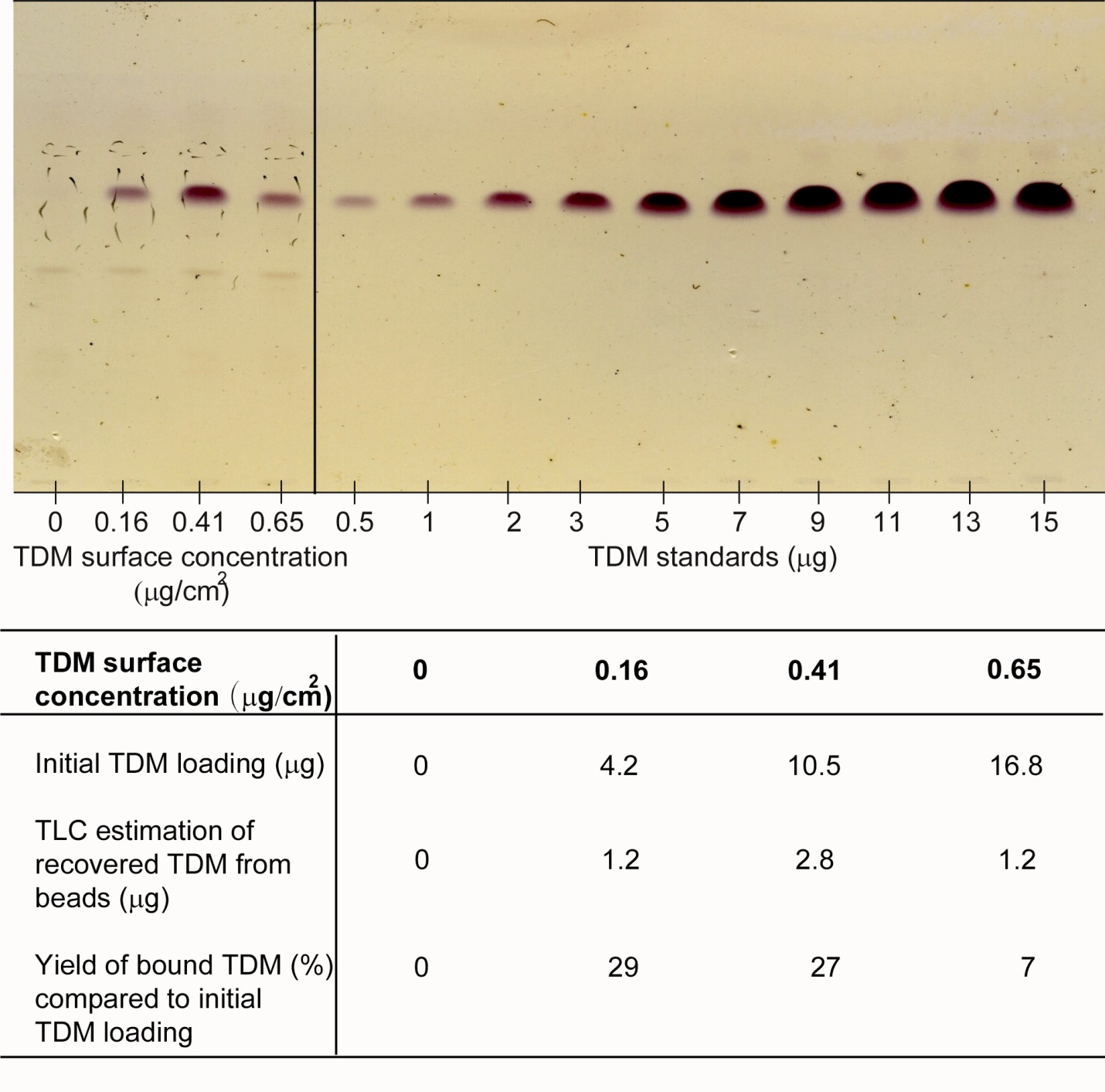


Figure S3. TLC analysis of TDM extracted from beads. TDM standards and extracted TDM from different bead preparations of a small batch (0.2mL) (*i.e.* with varying nominal TDM surface concentration, 0-0.65 µg/cm^2^) were spotted and TLC was carried out using CHCl_3_/CH_3_OH/H_2_O (65:24:4) as mobile phase and orcinol-based carbohydrate staining. A linear calibration curve of TDM standard was used to quantify the TDM present on each bead preparation. The table below represents varying TDM loading onto 4 × 10^7^ beads (total surface area: 25.6 cm^2^), the recovered bound TDM, and the % yield of bound TDM on each bead preparation.


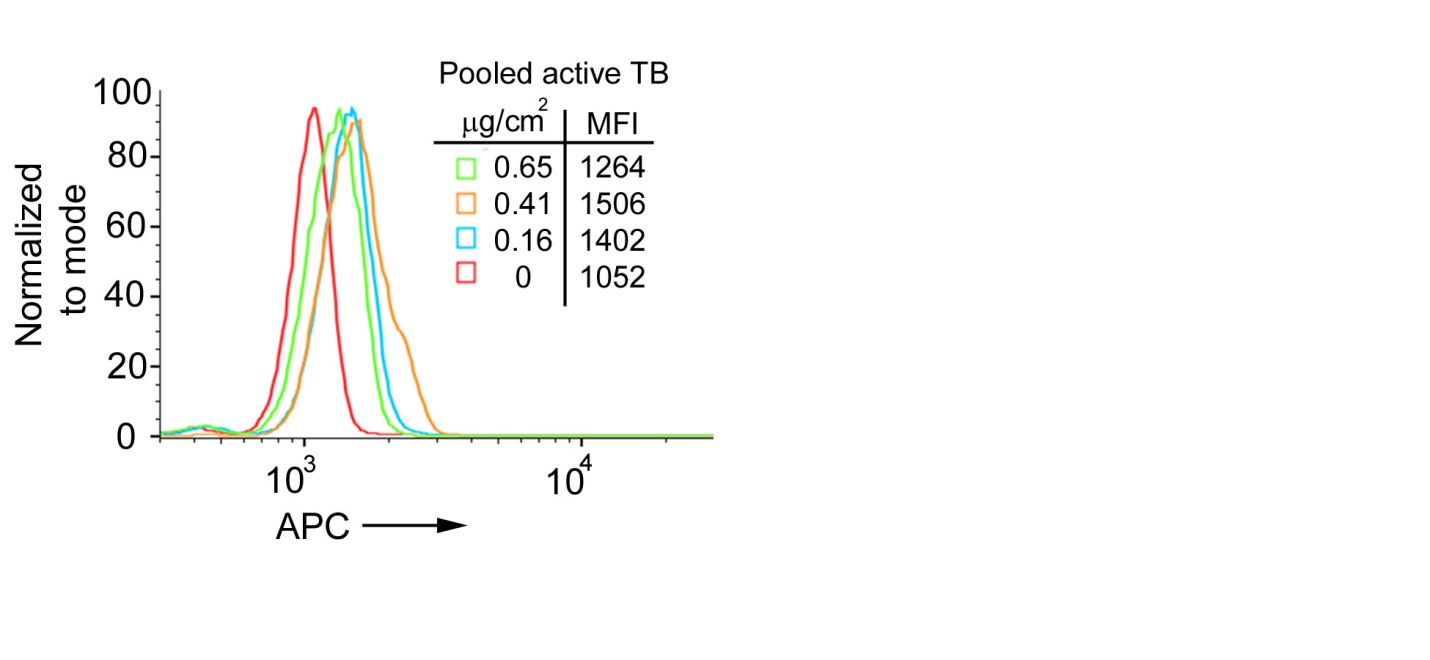


Figure S4. MB flow cytometry detection of anti-TDM IgG response in pooled ATB plasma (N=5) using beads coated with varying nominal surface TDM concentrations. Curves represent MFI peaks of TDM-coated MB preparations (0-0.65 μg/cm^2^) upon capture of anti-TDM antibodies from pooled ATB plasma, which was then stained with Alexa-647 conjugated secondary antibodies. Samples were acquired using flow cytometer (MACS quant analyzer).


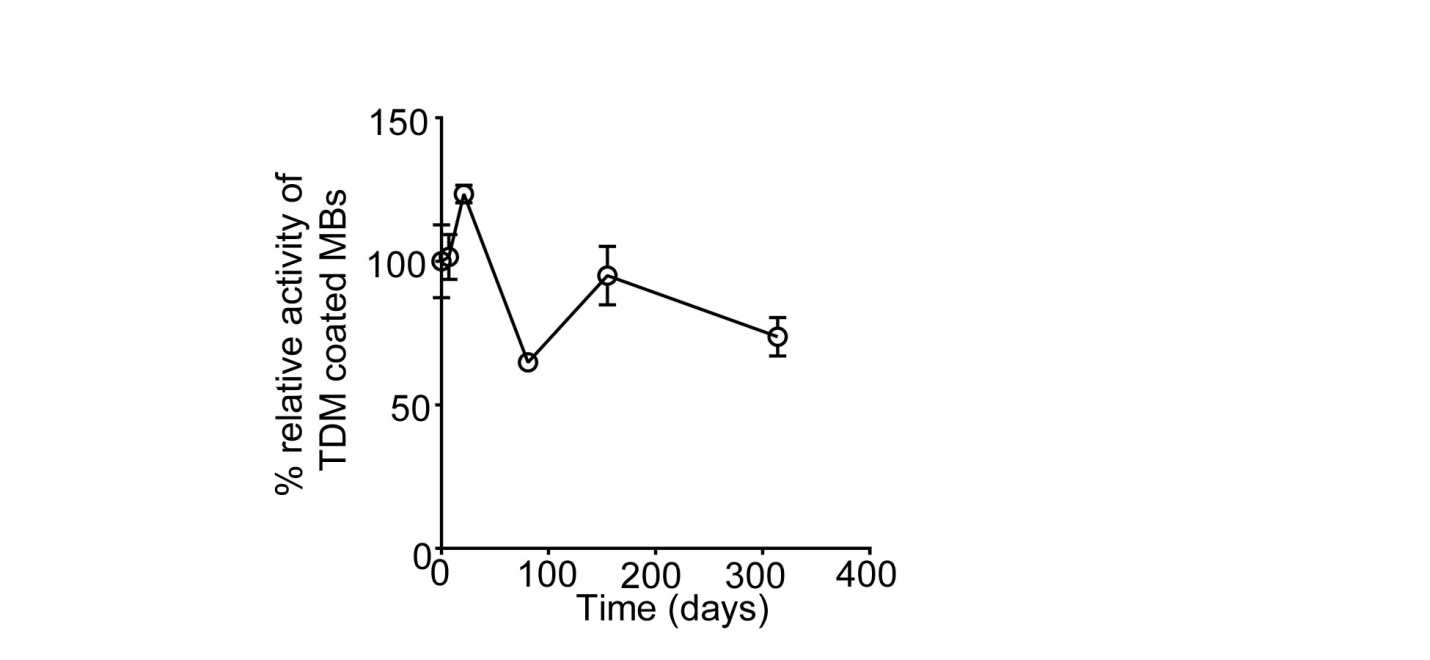


Figure S5. Stability of TDM-coated MBs (0.41 µg/cm^2^) at room temperature (22-25 ºC) over a period of 10 months. Anti-IgG response in a ATB patient plasma was measured using MTBE at different days, and % relative activity at time (t, days) was obtained from initial activity of beads at day zero. N=3


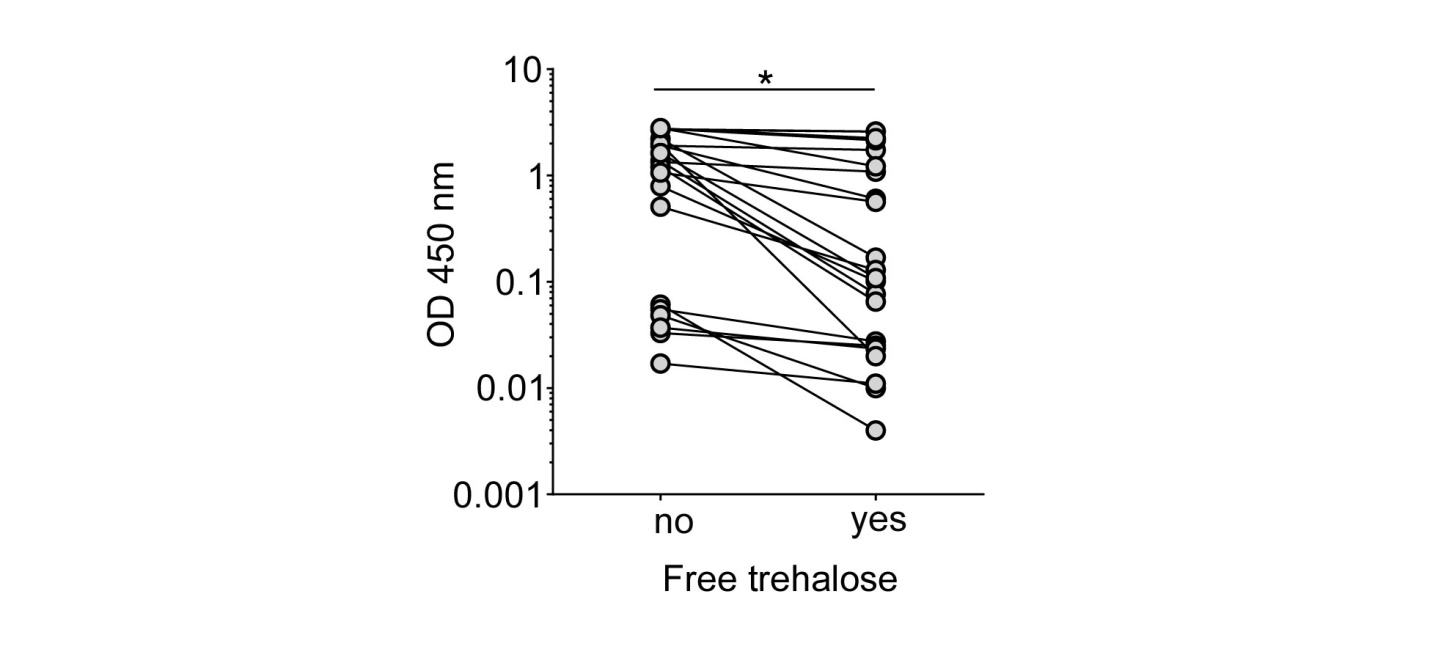


Figure S6. Competition of TDM plate ELISA using free trehalose. Anti-TDM IgG levels were estimated using conventional TDM plate ELISA, where plasma was pre-incubated with/without free trehalose (10%) for 1h prior to addition onto TDM-coated plates. N=22 (*, P=0.037).


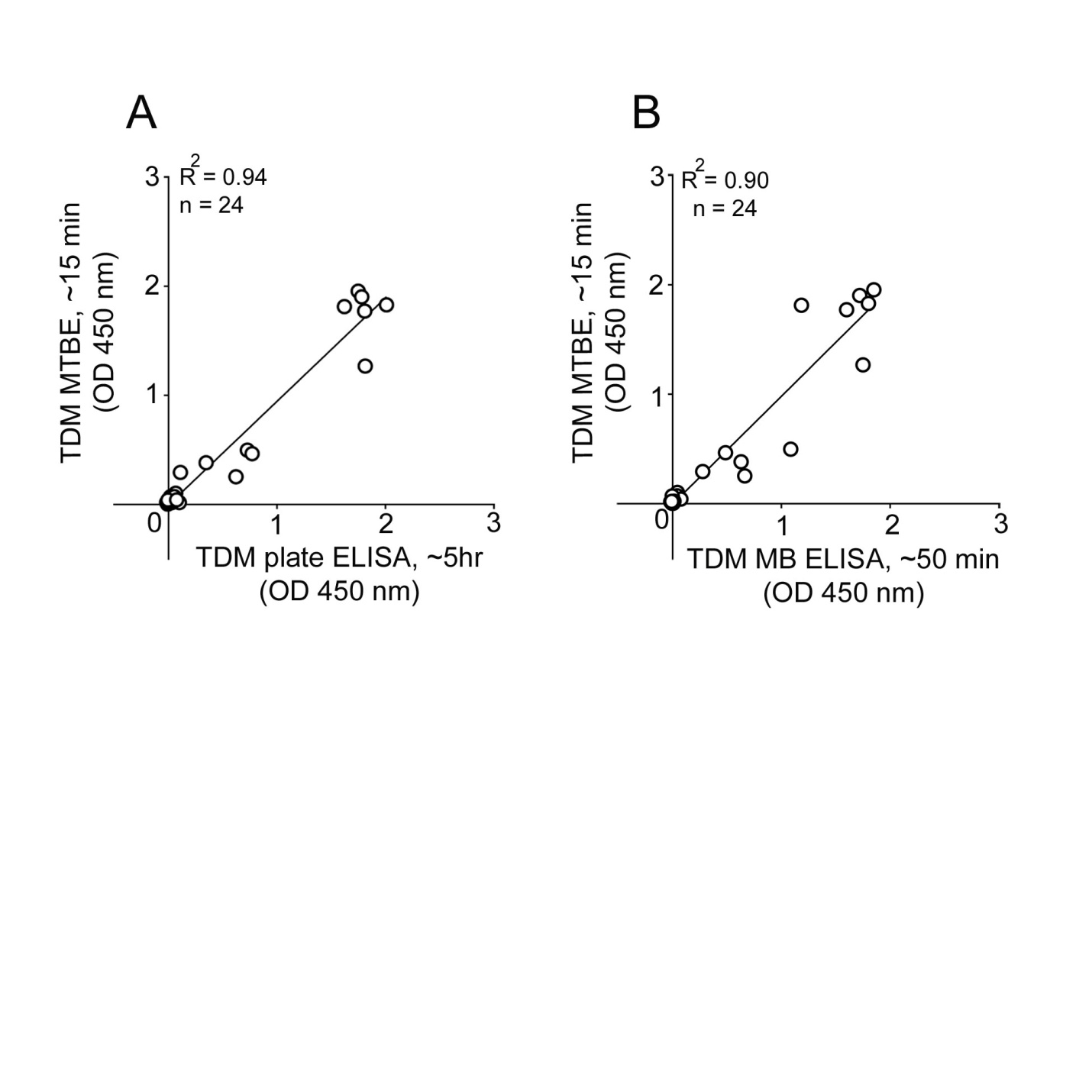


Figure S7. Correlation of anti-TDM IgG response between TDM MTBE. A) plate ELISA, B) MB ELISA for the same individuals. N = 24, ATB= 15; HC=9.


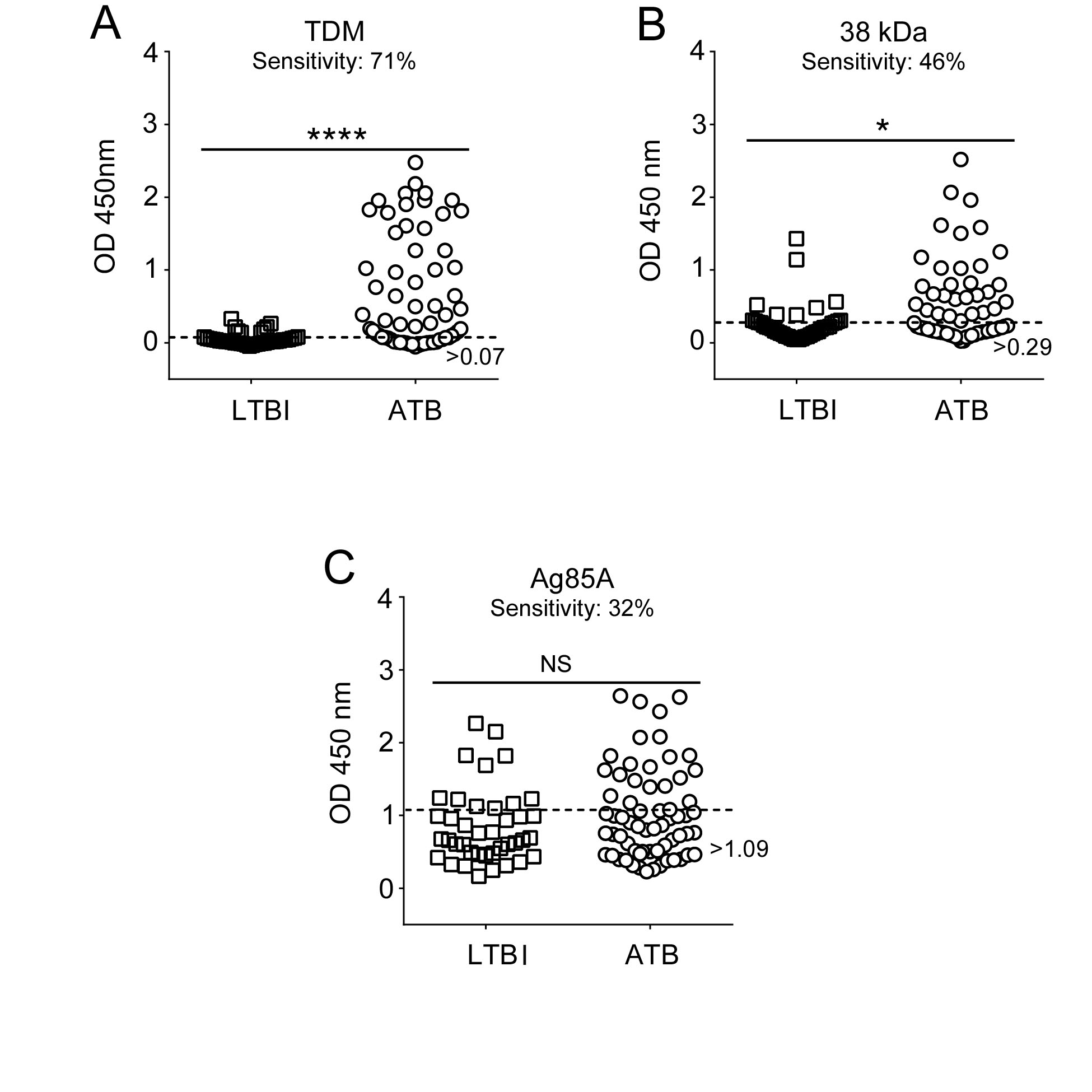


Figure S8. Distribution of IgG response against *Mtb* antigens. A) TDM, B) 38kDa, and C) Ag85A detected by MTBE to differentiate ATB patients from LTBI individuals. N= 105, ATB = 65, LTBI = 40 (****,P= <0.0001; *, P=0.035; NS = not significant).


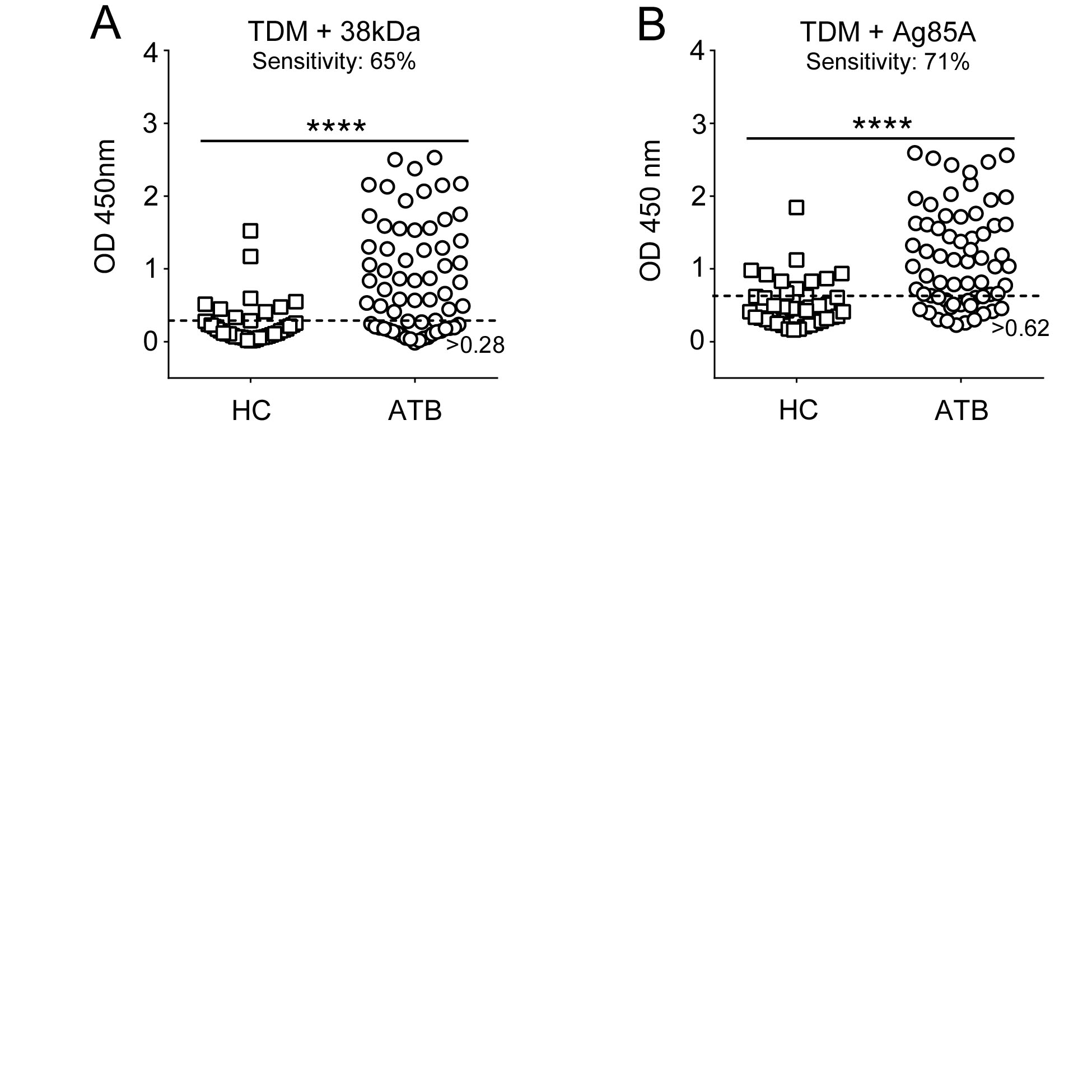


Figure S9. Distribution of collective IgG response against mixture of TDM with proteins detected using MTBE for differentiation of ATB and HC. A) TDM and 38kDa, B) TDM and Ag85A N= 106, ATB = 65, HC = 41 (****, P= <0.0001).


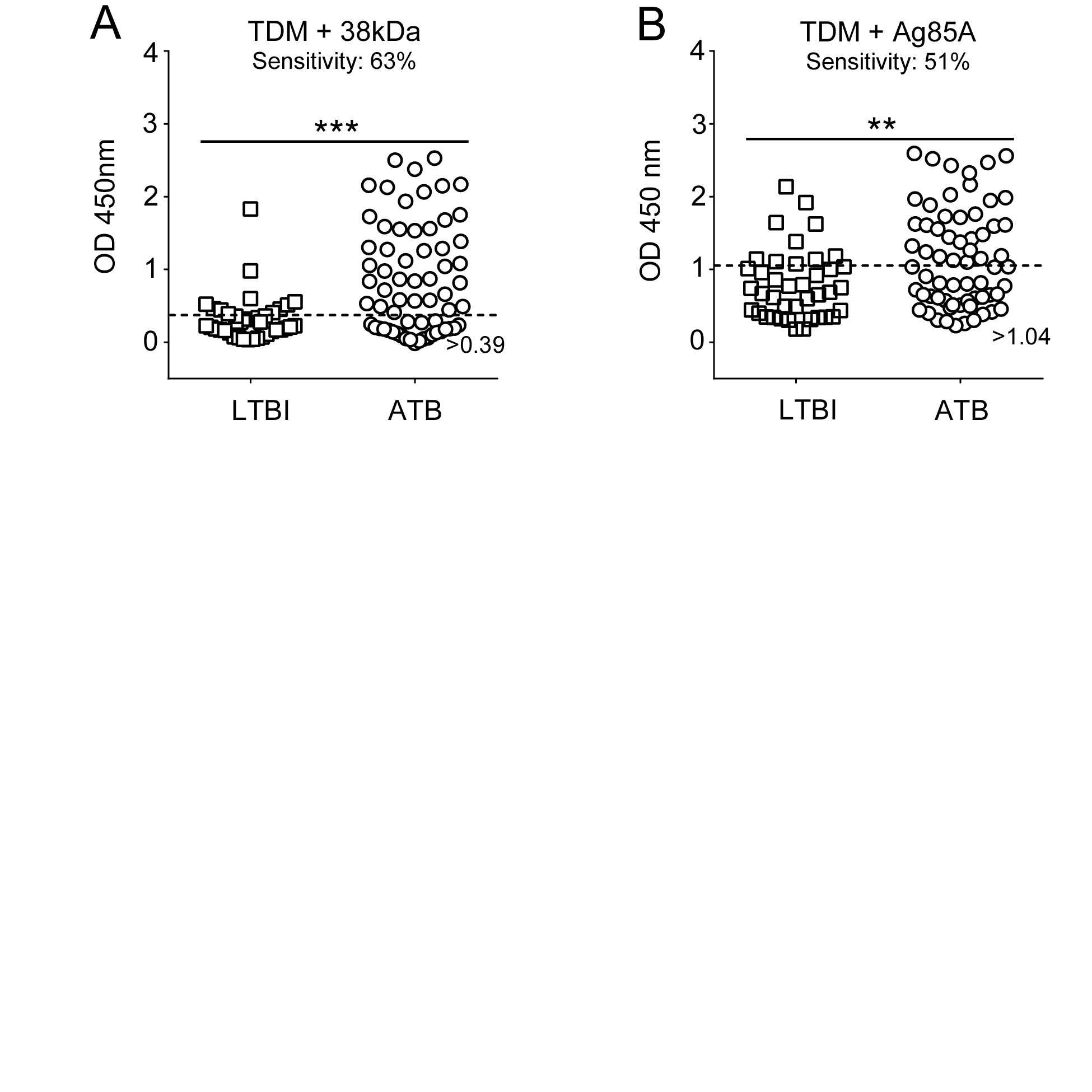


Figure S10. Distribution of collective IgG response against mixture of TDM with proteins detected using MTBE for differentiation of ATB and LTBI. A) TDM and 38kDa, B) TDM and Ag85A. N= 105, ATB = 65, LTBI = 40 (***,P=0.0003; **, P=0.007).


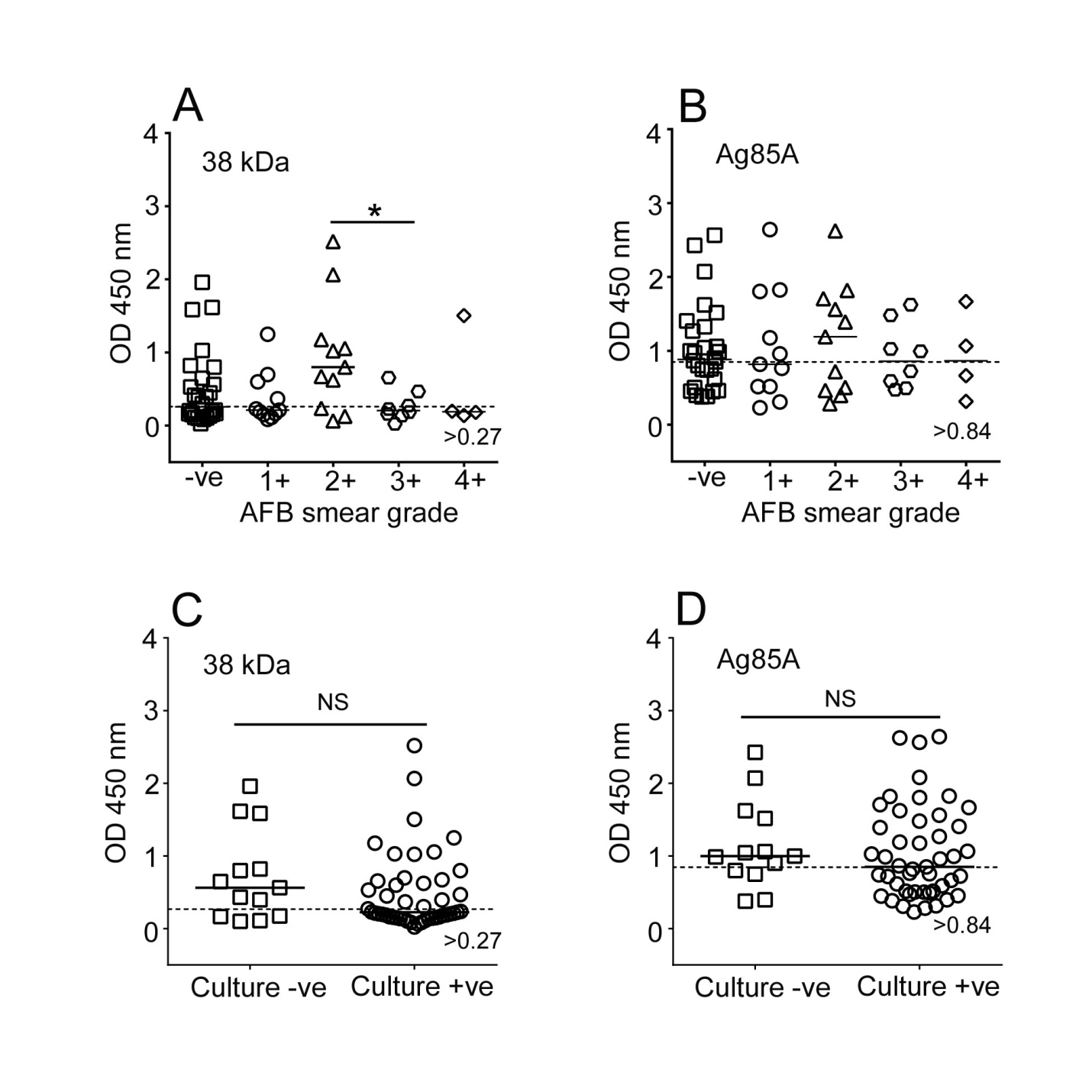


Figure S11. Distribution of anti-IgG response against 38kDa and Ag85A in ATB patients. A and B) AFB smear graded sputum samples. N = 62; -ve =28; 1+ = 11; 2+ = 11; 3+ = 8 and 4+ = 4), (38kDa; *, P=0.032). C and D) Samples stratified based on culture test. N=60; Culture positive samples N=47; Culture negative samples, N.=13. NS, not significant.

Supplementary Table

**Table S1.** Sensitivity and specificity comparison between conventional plate ELISA and MB TDM ELISA.

| **TDM-coated surface**  **(time of assay)** | **Sensitivity (%)** | **Specificity (%)** |
| --- | --- | --- |
| **MB ELISA (~ 50min)** | 68 | 75 |
| **Conventional plate ELISA (~5hr)** | 74 | 75 |

**SAtween these two test methods.000000000000000000000000000000000000000000000000000000000000000000000000000000000000000000000000**

Table S2. Evaluation of serodiagnostic potential of individual antigens and their combination using MTBE for the differentiation of ATB and LTBI individuals.

| **ATB vs. LTBI** | | | | | | | | |
| --- | --- | --- | --- | --- | --- | --- | --- | --- |
| **Antigen** | Sensitivity (%) | Specificity (%) | Positive predictive value (PPV) % | | | Negative predictive value (NPV) % | | ROC, AUC |
| **TDM** | 71 | 75 | | 82 | 61 | | 0.75 | |
| **38kDa** | 46 | 75 | | 75 | 46 | | 0.62 | |
| **Ag85A** | 32 | 75 | | 68 | 40 | | 0.57 | |
| **TDM + 38kDa** | 63 | 75 | | 81 | 57 | | 0.7 | |
| **TDM + Ag85A** | 51 | 75 | | 77 | 48 | | 0.65 | |

**Supplementary Methods**

**Characterization of TDM-coated MBs** Size distribution of TDM and BSA MBs were determined by dynamic light scattering (DLS) using Brookhaven particle size analyzer. For light microscope images, the MBs were dried on a 96 well plate, and were visualized using conventional microscope at 400× magnification.

TLC was used for estimation of amount of TDM bound to MB. TDM was extracted from the different MB preparation by sonication with chloroform-ethanol (1:1 v/v). The MB extracts were evaporated to dryness, and then reconstituted in chloroform-methanol and applied to silica gel TLC plate along with known amounts of TDM standards. For migration, chloroform-methanol-water 70:20:2 was used. After migration, bands were visualized by spraying an orcinol-sulfuric acid reagent followed by heating at 140 ºC for 30 s. This reagent reacts specifically with carbohydrate containing compounds.

For flow cytometry analysis, TDM-coated MBs and control BSA MBs, were first incubated with (1:200) pooled plasma of five ATB or HC samples (1 h reaction), followed by 3× buffer washing, and subsequently incubated with (1:1000) Alexa 647 labeled anti-IgG antibody for 1 h, which was further washed before acquiring MFI using flow cytometer (MACS quant analyzer). The data was analyzed using FlowJo software (Tree Star Inc.).
